# Insights into germline predisposition to pediatric lymphoid malignancies

**DOI:** 10.1038/s41375-025-02750-z

**Published:** 2025-09-09

**Authors:** Roshina Thapa, Kim E. Nichols, Richa Sharma

**Affiliations:** 1 Department of Oncology, St. Jude Children’s Research Hospital, Memphis, TN USA; 2 Cleveland Clinic Research, Cleveland, OH USA; 3 Case Western Reserve University, School of Medicine, Cleveland, OH USA

**Keywords:** Haematological cancer, Haematological cancer

## Abstract

Hematopoietic malignancies (HM) represent the most common form of pediatric cancer with lymphoid malignancies being the predominant subtype in kids. The majority of lymphoid malignancies are proposed to occur sporadically with environmental, infectious and inflammatory triggers impacting oncogenesis in ways that are not yet fully understood. With the increased adoption of germline genetic testing in children with cancer, genetic predisposition to lymphoid malignancies is now recognized as an important aspect of clinical care and research. Pathogenic variants in genes important for lymphocyte development, including cell differentiation, DNA recombination, recognition and repair of DNA damage, apoptosis, RNA processing, and intracellular signaling all converge on an increased risk for lymphoid malignancies. Herein, we review several genetic predispositions to lymphoid malignancies with a focus on the underlying biological defect, as well as the associated oncologic and non-oncologic manifestations.

## Introduction

Cancer predisposition syndromes (CPS) comprise a heterogeneous group of disorders associated with the presence of germline variants that confer a heightened risk of cancer. Individuals harboring CPS-related pathogenic variants may have a family history of cancer, characteristic physical features, and at times, non-oncologic manifestations. While CPS were once considered rare, recent large-scale genomic studies have revealed that up to 18% of children with cancer harbor germline pathogenic variants in a cancer predisposing gene [1–3].

Among childhood cancers, acute lymphoblastic leukemia (ALL) is the most prevalent pediatric malignancy, accounting for ~25% of cancer diagnoses in children under the age of 15 years [4]. Although the etiology of most lymphoid malignancies remains incompletely understood, increasing evidence highlights the contribution of inherited genetic factors [5–7]. To this end, identifying individuals with an underlying CPS is central to improving overall outcomes by enabling initiation of cancer surveillance and therapeutic interventions, as well as education and cascade testing of close relatives [8–10]. For example, individuals with certain CPS, such as DNA repair disorders, often require cancer treatment modifications to prevent therapy-associated organ toxicities, coupled with close monitoring for secondary neoplasms and management of non-oncologic manifestations. Beyond clinical management, knowledge of CPS-related genes and associated genetic pathways also deepens our understanding of the biology of lymphoid neoplasms, allowing for the development of novel treatment or preventive strategies.

In this review, we explore the genetic and cellular pathways central to lymphocyte development and examine how germline disruptions in these processes predispose individuals to lymphoid malignancies. By examining well-characterized syndromes, as well as newer “emerging” syndromes primarily associated with lymphoid cancers, we aim to provide an up-to-date overview of the current landscape in this evolving field.

## Lymphocyte biology and its perturbation in lymphoid malignancies

Hematologic malignancies arise from somatic and, in some cases, germline variants, often in conjunction with external factors, which disrupt the tightly regulated process of normal hematopoiesis. The rising cases of B-cell acute lymphoblastic leukemia (B-ALL) in recent decades, particularly in developed countries, suggests modern lifestyle and environmental factors as potential leukemogenic triggers [11–13]. Studies using mouse models have linked infectious and inflammatory triggers to an elevated risk of lymphoid malignancies [14–18]. However, the mechanisms by which environmental stimuli drive development of lymphoid neoplasms in predisposed individuals remain unclear and require further investigation. The complex interplay between genetic alterations and environment is underscored by studies demonstrarting that ~5% of healthy newborns harbor bona fide premalignant somatic alterations yet never develop lymphoid or myeloid malignancies in most cases [19, 20]. Given the critical role of the cell in which the first oncogenic hit takes place, we briefly review the developmental stages that occur during lymphocyte ontogeny and genes commonly disrupted in CPS that confer increased risk for lymphoid malignancies.

Hematopoietic stem cells (HSC) housed within the bone marrow (BM) give rise to a robust cellular system with the capacity to generate an estimated one million new erythroid and lymphoid cells per second in an adult human [21]. Integral to this differentiation cascade are transcription factors that drive specific blood cell lineage commitment and maturation [22–25]. Much of our understanding of these regulatory processes has been shaped by studies in human systems and mouse models, which unravel the functions of key transcription factors and hematopoietic lineage commitment. Originating from HSC, multipotent progenitors (MPP) differentiate into common myeloid progenitors (CMP) or early lymphoid progenitors (ELP). ELP give rise to early T-cell progenitors (ETP) or common lymphoid progenitors (CLP) [25]. ETP are committed to the T-cell lineage by Notch1 signaling factors, including T cell factor 1 (TCF-1), GATA3 and Bcl11b [26]. Specifically, GATA3 suppresses the differentiation of ETP into B- and myeloid cells, while Bcl11b restricts alternative fates by blocking gene expression (i.e. *Id2*, *PLZF*, and *Nfil3)* [27–29] critical for innate lymphoid and NK cell development. In the thymus, ETP undergo two stages of tolerance: positive selection, where T cells moderately binding self-MHC are retained, and negative selection, where T cells strongly binding self-antigens are eliminated to prevent autoimmunity [26].

The repression of FLT3 and activation of transcription factors such as TCF3, EBF, and PAX5 are key to committing CLP to the B cell lineage. Specifically, TCF3 and EBF coordinate B-cell lineage programming, with PAX5 driving final B-lineage commitment by activating B cell-specific genes and suppressing non-B cell genes [23, 24, 30]. Of note, a recent investigation that integrated functional analysis of distinct sorted B cell populations with single cell transcriptomics demonstrated that CLP retain myeloid potential, a finding that challenges our current understanding of lymphoid lineage restriction and could explain lineage promiscuity in some B-ALL cases [31]. Finally, pro-B cells initiate Ig gene rearrangement by RAG-1/RAG-2, regulated by IKAROS, to form the pre-B cell receptor (pre-BCR) [23, 24, 32, 33]. Immature B cells expressing functional B cell receptor (BCR) undergo tolerance testing in splenic germinal centers, further maturing into memory and plasma cells through somatic hypermutation, clonal expansion, and class switch recombination. Plasma cells migrate to the BM, while tissue-specific (i.e., resident) and circulating memory B cells produce antibodies upon re-exposure to antigens [34, 35].

**Fig. 1:**
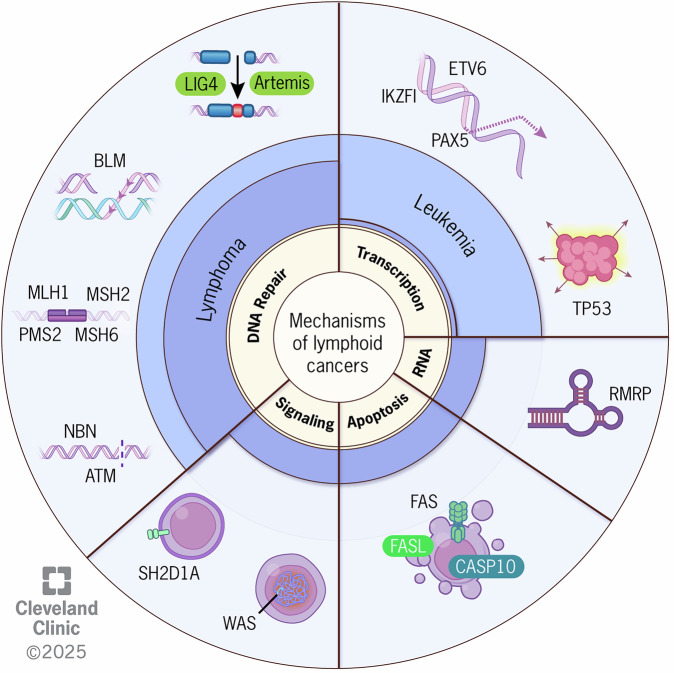
Germline deficiencies in key cellular processes predispose to pediatric lymphoid cancers. Susceptibility to lymphoid cancers in children results from heritable pathogenic variants affecting genes that encode proteins involved in critical pathways for lymphopoiesis including DNA repair, transcription, RNA biology, apoptosis, and cell signaling. MLH1 MutL homolog 1, MSH2 MutS homolog 2, MSH6 MutS homolog 6, PMS2 Postmeiotic segregation increased 2, BLM Bloom syndrome RecQ like helicase, NBN Nibrin, ATM Ataxia telangiectasia mutated, DCLRE1C DNA crosslink repair 1C, LIG4 ligase IV, IKZF1 Ikaros family zinc finger 1, PAX5 Paired box 5, ETV6 ETS Variant Transcription Factor 6, p53 protein p53, RMRP RNA component of mitochondrial RNA processing endoribonuclease, FAS FS-7-associated surface antigen, FASLG Fas ligand, CASP10 Caspase 10, WAS Wiskott-Aldrich syndrome, SH2D1A Src Homology 2 domain-containing protein 1A.

In summary, lymphopoiesis relies on a complex and coordinated network of factors involved in DNA replication, recombination, repair, cellular differentiation, and apoptosis. Recent studies on CPS have illuminated how heritable pathogenic variants in genes involved in these key cellular networks disrupt lymphocyte development, which heightens the risk for lymphoid malignancies. Disturbances in genes regulating early lymphoid development raise leukemia risk, while variants in genes governing later stages of B cell maturation cause an immunodeficient state generally associated with T/B cell dysregulation, poor tumor surveillance, opportunistic infections with oncogenic pathogens (e.g., EBV), and inaccurate repair of DNA damage resulting in high genomic instability and a predominance of lymphomas (Fig. 1).

In this review, we will focus on the biological and clinical features of well-established as well as emerging CPS associated primarily with an increased risk of lymphoid malignancies (Table 1). We refer to excellent reviews that discuss CPS [36, 37] associated with increased risk for myeloid malignancies or primary immunodeficiency [38–40].

**Table 1:** Classic and recent germline associations to lymphoid cancer predisposition in children.

Syndrome	Gene/Locus	Mode of inheritance	Non-tumor features	Hematologic cancer	Other neoplasms
Transcription factors associated disorders					
*ETV6-*associated leukemia predisposition (Thrombocytopenia V)	*ETV6* (12p13.2)	AD	Thrombocytopenia, bleeding, bruising	B-ALL, AML, CML, NHL, MDS, multiple myeloma	None
*IKZF1*-associated predisposition to B-ALL	*IKZFI* (7p12.2)	AD	Autoimmunity, immunodeficiency	B-ALL, T-ALL, NHL	None
Li-Fraumeni syndrome	*TP53* (17p13.1)	AD	None	B-ALL (hypodiploid), AML, MDS	Solid tumors (breast, soft tissue, bone, CNS)
*PAX5*-associated leukemia predisposition	*PAX5* (9p13.2)	AD	None	B-ALL	None
DNA repair disorders					
Ataxia telangiectasia	*ATM* (11q22.3)	AR	Cerebellar ataxia, telangiectasia, immunodeficiency, chronic lung disease	NHL, T-ALL, B-ALL, HL	Solid tumors (skin, GI, breast, CNS)
Bloom syndrome	*BLM* (15q26)	AR	Growth deficiency, immunodeficiency, rash, endocrine dysfunction, chronic lung disease	NHL, AML, ALL	Solid tumors (colorectal, breast, skin), GU
Constitutional mismatch repair deficiency	*EPCAM* (2p21) *MLH1* (3p22.2) *MSH2* (2p21) *MSH6* (2p16.3) *PMS2* (7p22.1)	AR	Café-au-lait spots	NHL (T cell > B cell), B-ALL	CNS (glioblastoma), GI (colorectal carcinoma)
NHEJ pathway deficiencies	*DCLRE1C* (10p13) *LIG4* (13q33.3)	AR AR	BMF, immunodeficiency, dysmorphic facies, SCID	B-NHL, EBV-associated B-NHL, MDS, AML	None
Nijmegen breakage syndrome	*NBN* (8q21)	AR	Microcephaly, unique facies, immunodeficiency	NHL, T-ALL, B-ALL, HL, AML	Solid tumors (CNS, rhabdomyosarcoma)
Primary immunodeficiencies and immune dysregulatory disorders					
Autoimmune lymphoproliferative and like disorders	*CASP10* (2q33.1) *FAS* (10q23.31) *FASLG* (1q24.3) *TET2* (4q24)	AD	Chronic lymphadenopathy, splenomegaly, cytopenias	HL, NHL	None
Cartilage hair-hypoplasia	*RMRP* (9p13.3)	AR	Short-limb dwarfism, fine hair, immunodeficiency, anemia, GI dysfunction	B-NHL	Basal cell carcinoma
Common variable immunodeficiency	*NFKB1* (4q24) *PIK3CD* (1p36.22) *STAT3* (17q21.2) *TNFRSF9* (1p36.23)	AD AD AD AR	Hypogammaglobulinemia, infections, autoimmunity, enteropathy, lymphoid hyperplasia	B-NHL, leukemia, HL	Gastric carcinoma,
Wiskott-Aldrich syndrome	*WAS* (Xp11.23)	XLR	Eczema, thrombocytopenia, bleeding, immunodeficiency	EBV-associated B-NHL, EBV-associated T-NHL, HL, AML, MDS	None
X-linked lymphoproliferative syndrome	*SH2D1A* (Xq25)	XLR	HLH, defective T- and NK-cell function	B-NHL	Aplastic anemia
Recent germline associations for lymphoid cancer predisposition					
*GAB2*	*GAB2* (11q14.3)	AD	None	B-ALL (hyperdiploid)	None
*LZTR1*	*LZTR1* (22q11.21)	AD	Noonan syndrome-like features	B-ALL (hyperdiploid)	None
*RUNX1*	*RUNX1* (21q22.12)	AD	Familial platelet disorder, inherited BMF	T-ALL, NHL, B-ALL, MDS/AML	None
*TYK2*	*TYK2* (19p13.2)	AD	None	T-ALL, B-ALL	None
*USP9X*	*USP9X* (Xq11.1)	X-linked, female-risk	Birth defects	B-ALL	None

*IKZF1* Ikaros family zinc finger 1, *B-ALL* B-cell acute lymphoblastic leukemia, *AD* autosomal dominant, *T-ALL* T-cell acute lymphoblastic leukemia, *NHL* non-Hodgkin lymphoma, *PAX5* paired box 5, *ETV6* ETS variant transcription factor 6, *AML* acute myeloid leukemia, *CML* chronic myeloid leukemia, *MDS* myelodysplastic syndrome, *BLM* bloom syndrome RecQ like helicase, *AR* autosomal recessive, *GU* genitourinary, *GI* gastrointestinal, *MLH1* MutL homolog 1, *MSH2* MutS homolog 2, *MSH6* MutS homolog 6, *PMS2* postmeiotic segregation increased 2, *CNS* central nervous system, *ATM* ataxia telangiectasia mutated, *HL* hodgkin lymphoma, *NBN* nibrin, *EBV* epstein-barr virus, *TP53* Tumor protein p53, *TNFRSF9* TNF receptor superfamily member 9, *PIK3CD* phosphatidylinositol-4,5-bisphosphate 3-kinase catalytic subunit delta, *NFKB1* nuclear factor kappa B subunit 1, *STAT3* signal transducer and activator of transcription 3, *B-NHL* B-cell non-hodgkin lymphoma, *FAS* FS-7-associated surface antigen, *FASLG* Fas ligand, *CASP10* caspase 10, *TET2* tet methylcytosine dioxygenase 2, *SH2D1A* src homology 2 domain-containing protein 1A, *XLR* X-linked recessive, *HLH* hemophagocytic lymphohistiocytosis, *NK* natural killer, *WAS* wiskott-aldrich syndrome protein, *RMRP* RNA component of mitochondrial RNA processing endoribonuclease, *TYK2* tyrosine kinase 2, *GAB2* GRB2-associated binding protein 2, *RUNX1* runt-related transcription factor 1, *BMF* bone marrow failure, *USP9X* ubiquitin specific peptidase 9 X-linked, *LZTR1* leucine zipper-like transcription regulator 1.

## Transcription Factors

### *ETV6-*associated predisposition (Thrombocytopenia 5)

The gene encoding ETS variant transcription factor 6 (*ETV6*, previously known as *TEL*), was first identified in 1994 through its involvement in the t(5;12) chromosomal translocation in chronic myelomonocytic leukemia [41]. *ETV6*, located on 12p13.2 locus, encodes the essential transcription factor ETV6 that plays a crucial role in embryonic development and hematopoiesis [42]. For example, *Etv6*^−/−^ mice are embryonic lethal due to defects in yolk sac angiogenesis as well as embryonal neural and mesenchymal populations [43] and conditional knockout studies reveal critical roles for ETV6 in maintaining HSC survival in the adult BM.

The first association between *ETV6* germline variation and B-ALL was in 2015 when four studies described carriers of *ETV6* variants with a median age of 11 years and clinical features of mild to moderate thrombocytopenia and 30% lifetime risk of hematologic malignancies, particularly B-ALL [44–47]. Germline damaging variants in *ETV6* are found in ~1% of children with B-ALL. In these cases, leukemic blasts are predominantly hyperdiploid (70%) with recurrent somatic alterations in *NRAS*, *KRAS* and *PTPN11* while the remaining 30% exhibit a diploid karyotype with frequent somatic *ETV6* and *PAX5* copy-number loss or inactivating mutations [48, 49]. B-ALL-associated germline *ETV6* pathogenic variants, which are evenly split between missense and truncating, cluster in the ETS DNA-binding domain. In vitro, mutant ETV6 proteins exhibit reduced DNA binding and decreased nuclear localization, properties associated with impaired repression of ETV6 target genes and putative dominant negative effects [48, 50]. A recent study assessing the functional effects of *ETV6* variants using CD34+ cell-derived megakaryocytes transduced with lentiviral particles encoding mutant ETV6 revealed disrupted expression of genes related to platelet biogenesis and cytoskeletal dynamics, such as CDC42 and RHOA, which supports the completely penetrant thrombocytopenia phenotype observed in these patients [51]. Murine studies modeling Thrombocytopenia 5-associated germlin*e Etv6* variants have shown reduced MPP4 mediated B cell development due to altered IL-18 and IL-13 secretion [52] and increased inflammatory gene expression among *Etv6*-mutated HSC [16].

### *IKZF1*-associated predisposition

IKAROS, encoded by *IKZF1* on chromosome 7p12.2, is the founding member of the Ikaros family of transcription factors [53, 54]. IKAROS features four N-terminal DNA-binding zinc finger motifs and two C-terminal C2H2 zinc-fingers that mediate DNA binding or dimerization with self or other IKAROS family members [55, 56]. Widely expressed in hematopoietic cells, IKAROS undergoes alternative splicing to generate multiple isoforms essential for regulation of pro-B to pre-B cell [57–59] and CD4 T cell differentiation [60]. The existence of loss of function or dominant negative *IKZF1* somatic mutations in high-risk B-ALL highlights IKAROS as an essential lymphoid tumor suppressor [61–64]. *IKZF*1 somatic alterations are also present in 1.2% of pediatric and 2.6-4.8% adult acute myeloid leukemia (AML) cases, underscoring its role in myeloid cancers [65–68].

Germline variation in *IKZF1* was initially described to modulate the risk of pediatric B-ALL through large genome-wide association studies which identified two common intronic variants [69, 70]. Subsequently in 2018, Churchman et al., identified heterozygous germline *IKZF1* variants co-segregating with disease in the members of a family characterized by autosomal dominant predisposition to B-ALL, B-lymphopenia and/or hypogammaglobulinemia and found a 0.9% prevalence of germline *IKZF1* variants in a large cohort of presumed sporadic B-ALL cases. Altogether, 28 unique *IKZF1* variants were identified in 45 individuals, raising the possibility that *IKZF1* represented a novel B-ALL predisposing gene [71]. Carriers of *IKZF1* pathogenic variants exhibit clinical features including immunological dysregulation (e.g., autoimmunity, immunodeficiency) with ~65–70% penetrance and a 10% risk of developing lymphoid malignancies, particularly B-ALL, by age 20 years and less commonly, T-cell acute lymphoblastic leukemia (T-ALL) and non-Hodgkin lymphoma (NHL) [9, 72]. *IKZF1* germline variants are distributed across the IKAROS functional domains and lead to dysregulated transcriptional activity and inefficient lymphoid maturation through haploinsufficiency, dimerization defects, dominant-negative, or gain-of-function effects [71, 72]. Recently, one new and two previously identified [70, 73] loci in *IKZF1* were found enriched among Hispanic/Latino children with B-ALL compared to children of predominantly European ancestry. It is possible that these variants contribute to the disproportionately higher incidence of B-ALL among Hispanic/Latino children [74]. Finally, the significance of germline variation in the Ikaros proteins for hematological phenotypes is highlighted by identifying germline pathogenic variants in the DNA-binding zinc fingers of *IKZF5*, which is linked to hereditary thrombocytopenia [75]. Taken together, these findings highlight the multifaceted role of IKZF1 and Ikaros family proteins in hematopoiesis, immune regulation, and leukemogenesis.

### Li-Fraumeni syndrome (LFS)

LFS is an autosomal dominant cancer predisposition syndrome caused by germline pathogenic variants in *TP53*, located at 17p13.1, with an estimated prevalence of 1 in 20,000 individuals [76, 77]. P53 remains one of the most studied tumor suppressor genes due to its critical role in maintaining genomic integrity. Wild-type p53 functions as a DNA-binding transcription factor, regulating genes involved in cell cycle arrest, apoptosis, senescence, DNA repair, and metabolism. Due to its indispensable functions, p53 is the most mutated gene in human cancers thereby promoting tumor progression, metastasis, and immune evasion [78].

Individuals with LFS are at increased risk for a variety of primary cancers, including breast cancer, soft tissue sarcomas, brain tumors, osteosarcoma, adrenocortical carcinoma, other solid cancers as well as hematologic malignancies, especially leukemia. The risk of leukemia in LFS patients is approximately six times higher than in the general population, occurring in about 3–5% of LFS cases with onset of leukemia under 45 years of age [79]. Hypodiploid B-ALL is the most common followed by therapy-related AML and myelodysplastic neoplasm. Low-hypodiploid B-ALL, defined by leukemia cells containing 32–39 chromosomes, is particularly associated with germline TP53 variants, found in up to 40% of cases [80]. In children with LFS, ALL often presents later (median age 15.5 years vs. 7.3 years in non-LFS cases) and with low leukocyte counts, distinguishing it from other ALL subtypes [79, 81]. LFS-associated hypodiploid ALL, associated with poor prognosis, exhibit TP53 loss of heterozygosity, along with alterations in *IKZF2* (65%) and RAS pathway genes (9%) [80, 82]. Therapy-associated ALL has also been described in a minor subset of breast cancer survivors with germline *TP53* variants [83]. One major contributor to poor survival in LFS is the high risk for second cancers with a 5-year cumulative incidence of 25% [81]. In response to the high penetrance for cancers in LFS, surveillance remains a cornerstone in the management of affected individuals [84].

### *PAX5*-associated predisposition

The paired box 5 (*PAX5)* gene, located on chromosome 9p13.2, encodes PAX5, an essential transcription factor that binds DNA through its conserved N-terminus paired box domain to activate B cell specific genes (e.g., *CD19*, *BLNK*) while repressing non-B lineage loci (e.g., *FLT3*, *CCL3*) [23, 85, 86]. PAX5 expression is exclusive to the B-lineage and modulated from the pre-pro-B cell stage to plasma cell differentiation [87]. The critical role of PAX5 in B cell development is made evident by the complete loss of B cells in *Pax5*^−/−^ mice [88]. Furthermore, the role of *PAX5* in leukemogenesis is demonstrated by the identification of somatic *PAX5* mutations in up to one-third of all B-ALL cases [89].

Germline heterozygous *PAX5* pathogenic variants are associated with predisposition to B-ALL showing incomplete penetrance and variable age of onset (e.g., 2–25 years) [90–96]. Five unique germline variants have been described thus far which are located in functional domains including octapeptide and N-terminal DNA binding domains as well as non-functional domains [90–95]. Proposed mechanisms of underlying predisposition to B-ALL include complete or partial loss-of-function and dominant negative effects [90, 92, 93, 97]. Acquisition of somatic alterations, in the remaining *PAX5* allele or mutations in *RAS* and/or *CDKN2A/B* were also observed in *PAX5*-associated B-ALL [90–96]. While these findings suggest a potential role for these somatic events in leukemic progression, their precise contribution remains to be fully elucidated, particularly in the context of other possible non-genetic contributors to leukemogenesis. The roles for environmental stressors, including infection [98] or downregulation of innate immunity [18], in B-leukemogenesis have also been demonstrated in *Pax5*^*+/–*^ mouse models. Interestingly, biallelic germline *PAX5* mutations were reported in a patient with clinical features of hypogammaglobulinemia, sensorimotor deficits and autism spectrum disorder. These disease features were recapitulated in a mutant mouse model harboring the patient specific mutations, suggesting new roles of *Pax5* in regulating neurodevelopment as well as B-cell development [99].

## Deficiencies in DNA replication, repair and cell cycle

### Ataxia telangiectasia (A-T)

The first description of individuals with A-T dates to 1926 by Syllaba and Henner, which precedes the classical description by Louis-Bar in 1941 [100]. Biallelic pathogenic variants in the ataxia-telangiectasia mutated (*ATM*) gene, located at 11q22.3 locus, were identified as the cause for A-T in 1995 [101]. *ATM* encodes a serine/threonine kinase that detects and repairs double strand break (DSB) by phosphorylating proteins like p53 and BRCA1 to regulate cell cycle, DNA repair, and apoptosis, to maintain genomic stability. ATM contains several key domains, including a PI3K-like kinase domain for DNA damage response, FAT C-terminal and breast cancer 1 C-terminal domain (BRCT) for protein interactions, an ATP-binding site for kinase activity, and a C-terminal domain that regulates ATM’s activation and function in DNA repair and genomic stability[102]. Over 3000 pathogenic/likely pathogenic *ATM* variants are described [103] across all functional domains with most resulting in reduced or absent ATM function. These variants disrupt DSB repair and impair meiotic recombination and antibody gene rearrangement during B-cell maturation [104, 105]. As a result, clinical manifestations of A-T include progressive cerebellar degeneration leading to ataxia, oculocutaneous telangiectasia, humoral and/or innate immunodeficiency, sinopulmonary infections, gonadal dysgenesis, radiosensitivity, and cancer predisposition [101, 106–108].

A-T affects 1 in 40,000 to 1 in 100,000 live births with ~1% of the population in the United States being *ATM* variant carriers. Individuals with A-T have a 10–30% risk of developing malignancies, particularly mature B-cell lymphomas [109–113]. In a large cohort of individuals with A-T and hematologic malignancies (*n* = 202), absent ATM kinase activity was correlated with a higher proportion of mature B-cell lymphomas, while residual ATM was linked to a predominance of T-ALL and other lymphoblastic malignancies, including T-cell lymphoblastic lymphoma (T-LBL), B-cell lymphoblastic lymphoma (B-LBL) and B-ALL[114]. Although hematologic malignancies are common, a recent study demonstrated that 26% of the primary malignancies among A-T individuals are solid tumors, which mostly occur after age 18 years [113]. In addition, individuals with A-T and cancer exhibited a 9-fold increase in standardized mortality ratio compared to non-cancer A-T individuals [113], likely due to a combination of treatment-related organ toxicities and non-standardized treatment regimens. However, the impact of therapy modification on survival outcome remains unclear as some studies note benefits such as reduced risk of death [113], reduced toxicities [115, 116] and comparable remission rates (T-ALL) [117, 118] but also inferior median survival [119]. Moreover, A-T associated co-morbidities including neurologic and advanced respiratory disease were independently associated with poor outcomes [114]. Large prospective studies are required to determine the impact of attenuated therapy on cancer outcomes in A-T.

### Bloom syndrome (BS)

BS is caused by biallelic pathogenic variants in *BLM*, located on chromosome 15q26, which encodes for the bloom syndrome protein (BLM) with ~300 reported cases worldwide. BLM, part of the RecQ helicase family, is an ATP-dependent helicase that associates with protein networks involved in DNA replication, repair, recombination, and mitochondrial homeostasis [120, 121]. BLM processes stalled replication forks to prevent formation of DSB and unstable secondary structures that lead to genomic instability. During homologous recombination (HR), BLM resolves Holliday junctions and facilitates strand exchange while suppressing excessive non-homologous end joining (NHEJ), a more error prone DSB repair pathway [121]. Disruption in *BLM* results in increased DNA breaks and chromosomal rearrangements and ultimately, a high rate of sister chromatid exchange, a classic finding in the cells of BS patients. Of note, this genome instability feature is also observed in individuals with Bloom-like syndromes caused by biallelic germline variants in *RMI1*, *RMI2* and *TOP3A* [121, 122].

Although rare, BS has a high incidence in the Ashkenazi Jewish population, which accounts for 25% of cases, due to a *BLM* c.2281delATCTGAinsTAGATTC p.(Tyr736fs) founder variant, present in 1% of Ashkenazi Jewish carriers [123]. BS-associated pathogenic variants are present throughout but concentrated in the helicase domain of the protein resulting in protein truncation or retaining full-length protein with loss of helicase activity [124]. Clinical manifestations of BS include growth deficiency, dermatologic rashes, feeding difficulties in infancy, endocrine disruption, chronic lung disease, immunodeficiency, and risk of early-onset cancers [122–124]. The Bloom Syndrome Registry reported 251 diagnosed malignant neoplasms in 155 individuals with BS, representing a cumulative cancer incidence of 52% by age 30 and 82.5% by age 40. NHL is the most common malignancy followed by colorectal and breast cancer. AML and ALL are also frequently observed with rare cases of Hodgkin lymphoma (HL). Individuals with BS are highly susceptible to developing secondary malignancies, with AML being the most prevalent. In addition, cancer therapy related toxicities are pronounced in BS patients with gastrointestinal complications being most common resulting in 30% of patients receiving modified therapy to mitigate side effects in one study [122].

### Constitutional mismatch repair deficiency (CMMRD)

CMMRD, first described in 1999, is a highly penetrant CPS with a prevalence of 1 in 1,000,000 live births. It is associated with biallelic germline variants in genes governing the mismatch repair (MMR) pathway, including *MLH1*, *MSH2*, *MSH6*, P*MS2* and *EPCAM* [125]. Genes in the MMR pathway correct mismatched bases and small insertion-deletion loops (indels) that occur during replication. Specifically, the MSH2/MSH6 heterodimer identifies base mismatches, while the MSH2/MSH3 heterodimer detects indels. Subsequently, the MLH1-PMS2 complex coordinates repair by recruiting exonucleases to remove the error-containing strand, followed by polymerase (*POLE*, *POLD1*) gap filling and ligation [126]. Monoallelic variation in MMR genes cause Lynch syndrome, an adult cancer predisposition syndrome characterized by colorectal and endometrial cancers. However, biallelic pathogenic variants in MMR genes, specifically, lead to CMMRD with a high risk for cancers and poor outcomes [127, 128].

A wide spectrum of cancers were recently described in a cohort of 201 CMMRD patients with 339 tumors, including brain (51%), gastrointestinal (22%), hematologic (18%) and other cancers (9%), affecting 90% of individuals by age 18 years and almost 100% by 40 years of age [128]. *MSH6* variants were most common, accounting for 65% of cases, followed by those affecting *PMS2* (25%), *MLH1* (6%), and *MSH2* (2.5%) with rare reports of *POLE* and *POLD1* variants as well as pathogenic variants in *EPCAM*, an epigenetic silencer of MSH2 [128], [129]. Due to the highly error-prone MMR machinery inherent to CMMRD, the associated tumors have exceptionally high tumor mutation burden (TMB) and microsatellite instability, making them amenable to checkpoint inhibitor therapies [130–134]. Interestingly, of the three tumor subtypes, CMMRD-associated hematologic malignancies have the lowest TMB and lack enrichment for *TP53*, *RAS-MAPK* pathway and *ATRX* mutations, which are noted in CMMRD-associated solid tumors. Hematological cancers accounted for 18% of all malignancies, with T-cell lymphoma and leukemia representing the majority (50%) of cases. This was followed by mature B-cell lymphoma (23%), precursor B-cell leukemia (15%), and myeloid leukemia (8%). Less frequent subtypes included mixed phenotypic leukemia and Hodgkin lymphoma, each comprising 2% of hematological cancers [135]. Individuals with hematologic malignancies have a 67% 10-year survival, which is better than for those with CNS tumors with survival of 39%. The low surveillance detection rate of 16% for hematologic malignancies highlights the need for better surveillance methods in this highly penetrant CPS.

### NHEJ pathway deficiencies

NHEJ is a critical but error-prone DSB repair pathway associated with rare (1 in 1,000,000 live births) germline alleles that predispose to heightned risk for lymphomas. One major role of NHEJ is to orchestrate VDJ recombination required for generating T and B cell receptors and immunoglobulins [10, 136]. During this recombination event, *LIG4*, which encodes DNA ligase IV, ligates the final DSB. In the presence of abnormal Ligase IV function, as seen in individuals with Ligase IV deficiency due to biallelic *LIG4* germline variants, incomplete NHEJ leads to accumulation of DSB in neurons and HSC causing cellular mutagenesis and apoptosis [137, 138]. Patients present at variable ages with microcephaly, growth retardation, developmental delay, dysmorphic facial traits, varying degrees of immunodeficiency, pancytopenia, radiosensitivity, and increased susceptibility to malignancies [138]. Bone marrow failure (BMF) is the most common hematologic manifestation followed by B-lymphoid malignancies. AML and myelodysplastic neoplasm have also been described [10, 138]. Most recently, autosomal dominant LIG4 haploinsufficiency due to monoallelic *LIG4* pathogenic variants were demonstrated in individuals with immune dysregulation [139] but without cancer risk. Biallelic *LIG1* variants have also been described in patients with hypogammaglobulinemia, lymphopenia, high γδT cells, and erythrocytic macrocytosis without an elevated cancer risk described thus far [138, 140]. Biallelic pathogenic variants in another NHEJ gene, *DCLRE1C*, which encodes ARTEMIS, has been identified in patients with severe combined immunodeficiency, radiation sensitivity and increased predisposition for EBV-associated B cell lymphomas [141]. These studies highlight the important role of NHEJ pathway in lymphopoiesis.

### Nijmegen breakage syndrome (NBS)

NBS was named after the Dutch city, Nijmegen, where the c.657_661del5 founder variant in *NBN* on chromosome 8q21 was first identified in 1981 [142, 143]. Although the estimated prevalence is ~1 in 100,000 live births worldwide, NBS is most common in individuals of Eastern European/Slavic descent due to the founder germline c.657_661del5 variant, which has an estimated carrier frequency of 1 in 155 in these populations [143–147]. *NBN* encodes for nibrin, which facilitates DNA damage response through a forkhead-associated (FHA) domain for binding phosphopeptides and a BRCT domain for protein-protein interactions. Nibrin, part of the MRN complex, detects DSB, aids ATM activation and regulates processes that require DSB like HR, NHEJ, V(D)J recombination and immunoglobulin class switching in B- and T-cells [148–151].

NBS is an autosomal recessive disorder characterized by microcephaly, unique craniofacial features, growth retardation, combined immunodeficiency, recurrent sinopulmonary infections, radiosensitivity, and an increased risk of lymphoid malignancies [152–154]. Unlike A-T that incurs up to 30% lifetime cancer risk, a natural history study of 241 NBS patients across 11 countries demonstrated almost 80% cumulative incidence of cancer by the age of 20 [153]. Ninety percent of cancers are of hematologic origin with NHL comprising 63% of tumors followed by acute leukemias (21%, mostly T-ALL), HL and other solid malignancies [153]. Malignancies are the major cause of death in NBS patients with poor prognosis due chemotherapy-related toxicities, immunodeficiency, and lacking standardized treatment regimens [155]. In contrast to the historically poor outcomes in A-T individuals following bone marrow transplantation (BMT) for relapsed/progressive malignancy, NBS patients with cancer who received BMT have improved outcomes and a higher 20-year overall survival compared to NBS patients who were not transplanted [153]. The impact of *NBN* heterozygous pathogenic variants on cancer risk continues to be a moving target with a recent study in adults suggesting these carriers to exhibit a potential pan-cancer risk [156]. Most recently, a study of 4,325 pediatric B-ALL patients identified 25 unique, putatively damaging *NBN* variants in 50 patients, showing a significant overrepresentation compared to noncancer controls. Although functional assays confirmed 14 variants to be loss-of-function, these carriers had similar survival outcomes to those with wild-type NBN, suggesting they can safely receive B-ALL therapy [157].

## Primary immunodeficiencies and immune dysregulatory disorders

### Autoimmune lymphoproliferative syndrome (ALPS) and ALPS-like disorders

ALPS is caused by defective signaling in the apoptosis pathway resulting in inefficient removal of autoreactive T cells and overall immune dysregulation. Mono- or biallelic germline variants in *FAS* (locus 10q23.31, encodes CD95), *FASL*, (locus 1q24.3, encodes CD95L) and *CASP10* (locus 2q33.1, encodes caspase 10), can cause different subtypes of ALPS. The shared diagnostic criteria among ALPS subtypes include elevated peripheral double-negative T (DNT) cells, chronic lymphadenopathy and/or splenomegaly as a result of lymphoproliferation, chronic multilineage cytopenias, and risk for lymphomas [158], particularly later in the disease course. Although ALPS is associated with common genetic defects, the disease exhibits considerable variability in symptoms and characteristics across individuals and subtypes, resulting in underdiagnosis and misdiagnosis in many cases[159]. For this reason, precise prevalence and incidence of ALPS are unclear. The risk of developing HL and NHL is increased 51- and 14-fold and can be EBV-associated [159, 160]. Recently, two individuals with ALPS-like features developed T-cell lymphoma and were found to have novel germline *TET2* variants. As part of the Tet family, TET2 protein and its effectors coordinate DNA methylation/demethylation in hematopoietic cells and as such is one of the commonly mutated genes in hematopoietic cancers, especially myeloid cancers. Of these two cases, one exhibited biallelic compound heterozygous variants while the other patient harbored a monoallelic *TET2* variant [161]. ALPS-associated lymphomas respond well to conventional chemotherapy but disease monitoring during remission can be challenging due to the underlying proliferation phenotype [162].

### Cartilage hair hypoplasia (CHH)

CHH is an autosomal recessive disorder caused by biallelic pathogenic variants within the RNA component of mitochondrial RNA processing endoribonuclease (*RMRP)*. Located at chromosome 9p13, *RMRP* encodes a non-coding RNA, which is involved in mitochondrial RNA processing, ribosome biogenesis by processing the 5.8S rRNA, regulating G1/S phase of the cell cycle by interacting with major proteins like pRB, mitigating cellular stress response, and telomere regulation [163, 164]. Although a rare syndrome in the general population, CHH is enriched in the Amish and Finnish populations, with respective incidence of 1 in 1340 and 1 in 23,000 live births [165]. Over 130 CHH-associated variants in *RMRP* have been described, with the c.71A>G variant being the most common in Amish and Finnish. Most variants are found in the transcribed region, with additional variants, deletions, and duplications identified in the promoter region [166, 167]

The phenotype of CHH is highly variable and includes disproportionate short stature, joint hypermobility, fine silky hair, immunodeficiency, anemia, gastrointestinal dysfunction, impaired spermatogenesis, and increased risk for malignancy, particularly NHL and basal cell carcinoma [165, 168]. Kukkola et al., reported 16 cases of lymphoma, with diffuse large B-cell lymphoma (DLBCL) as the most common subtype, among 160 Finnish patients diagnosed at a median age of 26.4 years. All but one patient were treated with standard chemotherapy regimens and no therapy related infections were reported. However, the mortality rate was dismal at 69% and proposed to be due to advanced stage disease at presentation [169]. The high risk for lymphoid malignancies with poor outcomes in CHH individuals presses the need for studies that explore the mechanistic underpinning of CHH and address therapeutic strategies and surveillance.

### Common variable immunodeficiency (CVID)

CVID is the most prevalent primary immunodeficiency with an incidence of 1:25,000 to 1:50,000 and has significant genetic and phenotypic heterogeneity [170, 171]. Due to increased genetic sequencing, 25–30% of CVID patients have an identifiable germline defect in genes required for B-cell development and function [172, 173]. Since immune dysregulation is a major feature, there is a high risk of hematologic and solid malignancies in older CVID individuals with a prevalence of 10% (range of 1.5–20.7%) [174–180]. B-NHL is the most common malignancy reported (usually EBV negative) followed by gastric carcinoma, leukemia, HL, and other cancers. Interestingly, studies have observed an association between increased risk of malignancy and elevated IgM [174, 176, 181], which could serve as a potential maker for lymphoma. Monogenic CVIDs with highest predisposition to cancer include the activated phosphoinositide 3-kinase delta syndrome (APDS), nuclear factor kappa B subunit 1 (NF-kB1) insufficiency, and signal transducer and activator of transcription 3 (STAT3) gain-of-function [182–184]. Homozygous variants in *TNFRSF*9 (tumor necrosis factor receptor super family member 9), which encodes the costimulatory immune checkpoint CD137/4-1BB essential for T- and B-cell function, have also been described to cause CVID-like disease [185–187]. Moreover, an early predisposition to EBV-associated lymphoma, has also been noted [187].

### Wiskott–Aldrich syndrome (WAS)

WAS is a X-linked syndrome initially described by Dr. Wiskott in 1937, in a report of a family with several members who had eczema, thrombocytopenia and recurrent infections. This was followed by a similar but independent clinical description of immune dysfunction and platelet abnormalities in a family in 1954 by Dr. Aldrich. Named after both physicians, Wiskott-Aldrich Syndrome is caused by hemizygous alterations in the *WAS* gene, which was mapped to Xp11.23 in 1994 and encodes WAS protein (WASp) [188]. WASp, primarily expressed in hematopoietic cells, has a complex structure with 7 domains that regulate actin cytoskeleton reorganization by activating the actin-related protein 2/3 (ARP2/3) complex for processes such as cell movement, vesicular trafficking, and pathogen infection [189]. WASP is crucial for proper T-cell and NK-cell function, antibody production, and chemokinesis [190].

WAS affects 1–10 male newborns per 1,000,000 and typically presents in infancy with features of micro-thrombocytopenia, leading to intermittent mucosal bleeding, petechiae and purpura, eczema, immunodeficiency resulting in recurrent bacterial and viral infections, autoimmunity (40%) and increased risk of cancer [188, 190]. Approximately 13% of WAS patients develop malignancies, with an average age of onset of 9.5 years. The most common cancer subtype is EBV-associated B-NHL, which frequently arises in extranodal sites such as the brain, lungs, or gastrointestinal tract. EBV-associated T-NHL and HL have also been reported [191]. Over 300 pathogenic variants in the WAS gene have been identified, with approximately one-third of these mutations occurring in nine mutational hot spots [192]. Complete loss-of-function variants (e.g., nonsense alterations, small indels or larger deletions) typically lead to severe clinical manifestations associated with higher mortality due to infections, hemorrhage, and/or cancers [193, 194]. In contrast, hypomorphic variants result in X-linked thrombocytopenia, a milder phenotype of WAS associated with thrombocytopenia and a lower occurrence of malignancy [195]. Gain-of-function variants lead to AML/myelodysplastic neoplasm and X-linked neutropenia [196].

### X-linked lymphoproliferative syndrome (XLP)

First described in 1975 by Purtilo et al. [197], X-linked lymphoproliferative syndrome type 1 (XLP1) is a rare X-linked immunodeficiency caused by germline variants in the gene *SH2D1A* with an incidence of 1 in every 1,000,000 – 2,000,000 male individuals [198]. *SH2D1A*, located on chromosome Xq25, encodes the signaling lymphocytic activation molecule (SLAM)-associated protein (SAP), a small SH2 domain containing signaling protein that binds to tyrosine residues present within the receptors of the “SLAM” family (SLAM, Ly9, 2B4, CD84 and NTB-A), which are expressed on hematopoietic and immune cells. By blocking the binding of inhibitory phosphatases and facilitating recruitment of the tyrosine kinase Fyn, SAP enables the generation of downstream signaling that is critical for CD4+ T cell Th2-type cytokine production, CD8+T and NK cell cytotoxicity, and iNKT cell development [199]. Since most XLP1-associated *SH2D1A* pathogenic variants reduce or eliminate SAP expression, it is believed that the manifestations of SAP deficiency occur due to impaired SLAM receptor signaling on T, NK, and iNKT cells [200].

The clinical features of XLP syndrome include EBV-induced hemophagocytic lymphohistiocytosis (previously known as “fulminant infectious mononucleosis”), dys- or hypogammaglobulinemia, and elevated risk of B-lymphoproliferative disorders, lymphomas (~50% EBV-related) and aplastic anemia [199, 201]. Lymphomas occur in ~30% of patients and include high-grade NHL, primarily of B-cell origin (82%) that are often extranodal [202]. The average age at lymphoma diagnosis is 15 years (ranging from 2 to 40 years), and secondary lymphomas are infrequently observed [200].

## Discovery of new germline conditions predisposing to lymphoid cancers

The increased accessibility of high throughput sequencing has led to the recent identification of additional lymphoid cancer predisposition genes. Rare germline variants in *GAB2*, a scaffold protein that mediates signaling through the PI3K/AKT and the ERK/MAPK pathways, were identified in three of 57 cases of high-hyperdiploid ALL [203]. In a cohort of pediatric B-ALL patients, ~2% harbored damaging germline variants in *LZTR1*, a gene more commonly associated with Noonan syndrome. These variants were associated with development of hyperdiploid karyotype B-ALL, even in the absence of Noonan syndrome-like features. *LZTR1*, located on chromosome 22q11.21, encodes a protein that regulates the RAS-MAPK pathway by facilitating RAS ubiquitination and degradation. In a Drosophila model, these *LZTR1* pathogenic variant demonstrated RAS pathway activation, ERK accumulation, cell proliferation, and apoptosis [204].

Although the role of RUNX1 in normal myelopoiesis and its germline alterations resulting in predisposition to familial myelodysplastic neoplasm/AML disorders is well known [205–208], recent studies illuminate the critical role that RUNX1 plays in T-ALL cell development. Specifically, germline sequencing of children with newly diagnosed B-ALL and T-ALL identified enrichment for *RUNX1* variants in T-ALL which were primarily loss-of-function leading to reduced RUNX1 transactivation activity[209]. Cases of T-LBL have also been described in germline *RUNX1* pathogenic variant carriers [208, 210]. Gain-of-function pathogenic variants in *TYK2*, encoding tyrosine kinase 2, were identified in two individuals with pediatric ALL [211]. Lastly, X-linked *USP9X* encodes ubiquitin-specific peptidase 9 protein, which is critical for protein stability, cellular signaling and development was recently identified as a novel female-specific leukemia predisposition gene among a cohort of probands and family members with multiple birth defects and cancer. Interestingly, *USP9X* is somatically mutated in sporadic B-ALL but without a sex-bias [212]. These recent studies suggest that the search for lymphoid malignancy predisposing genes is not over, and future studies will likely enhance our understanding of the germline genetic factors contributing to the risk for lymphoid cancers.

## Impact of germline information on treatment decisions

Knowledge of an underlying predisposing condition for lymphoid malignancies is crucial for guiding treatment decisions that minimize the risk for secondary neoplasms, particularly in individuals with mutations affecting the DNA damage response pathway. For example, individuals with A-T, NBS and BS often require cancer therapy modifications to prevent organ toxicities and require frequent surveillance for development of secondary neoplasms [10, 114, 122, 153]. The addition of such targeted therapies offers the potential to reduce or even eliminate the need for toxic chemotherapy agents, which can contribute to late-effects and chronic health conditions as well as secondary cancers. One such example includes the use of the bi-specific T cell engager blinatumomab, which exhibits therapeutic efficacy in the treatment of childhood B-ALL [213, 214]. Another reason for early identification of genetic predisposition is the enabling of proactive screening programs, aimed at the early detection and management of new cancers (most commonly solid tumors) as well as non-cancer features, which are common in patients with some of the syndromes discussed.

When considering genetic testing in a child with lymphoid malignancy, it is essential to obtain samples from non-hematopoietic tissue (e.g., cultured skin fibroblasts) to avoid confounding results due to contaminating malignant cells in blood samples or saliva. Additionally, any associated functional tests, such as telomere length analysis, chromosomal breakage studies, lymphocyte subset and T-cell proliferation assays, should ideally be performed prior to the initiation of radio-and/or chemotherapy, as these treatments can alter test results and complicate interpretation. Comprehensive guidelines for genetic evaluation and long-term follow-up, such as those outlined in an ASCO expert review [215], will be essential in translating these findings into improved patient care and survivorship planning.

For several lymphoid cancer predisposition syndromes, including LFS, DNA repair deficiencies and most primary immunodeficiency disorders, allogenic BMT remains the standard for treatment of progressive/relapsed disease. Preventative transplants in the case of NBS or certain myelodysplastic neoplasms, BMF conditions or PIDs can also be considered [153, 216]. In these cases, careful consideration must be given to selecting family donors who do not carry the same genetic variant as the patient, since transplanting cells from a donor with the same genetic defect could increase the risk of transplant-related complications and donor-derived hematopoietic malignancy. Radiosensitivity disorders, like A-T and BS [217, 218] require further investigation to determine an effective conditioning and post-transplant maintenance regimen to strike the tough balance between toxicity and antitumor effect to achieve disease cure.

## Summary and future directions

Significant progress has been made in elucidating the hereditary basis of lymphoid malignancies, advancing our understanding of disease biology and paving the way for the development of novel and more effective treatment and surveillance strategies. However, several gaps remain unresolved. The full spectrum of genetic factors is not fully defined, and many germline variants remain of uncertain clinical significance. Given that the treatment of most DNA repair disorders associated lymphoid malignancies continues to be a challenge due to high incidence of organ toxicity, there is a pressing need for more sensitive methods of early cancer detection, as well as effective and less toxic therapies that are targeted to underlying disease mechanisms. Importantly, establishing a universal and standardized therapy approach among these rare diseases poses further challenges. To tackle these issues and improve outcomes for affected individuals, multi-institutional and multi-disciplinary collaboration is necessary.
